# Elucidating the contributory role of microRNA to cardiovascular diseases (a review)

**DOI:** 10.1016/j.vph.2018.10.010

**Published:** 2019-03

**Authors:** Jason L. Johnson

**Affiliations:** Laboratory of Cardiovascular Pathology, Bristol Medical School, University of Bristol, UK

## Abstract

Cardiovascular diseases encompassing atherosclerosis, aortic aneurysms, restenosis, and pulmonary arterial hypertension, remain the leading cause of morbidity and mortality worldwide. In response to a range of stimuli, the dynamic interplay between biochemical and biomechanical mechanisms affect the behaviour and function of multiple cell types, driving the development and progression of cardiovascular diseases. Accumulating evidence has highlighted microRNAs (miRs) as significant regulators and micro-managers of key cellular and molecular pathophysiological processes involved in predominant cardiovascular diseases, including cell mitosis, motility and viability, lipid metabolism, generation of inflammatory mediators, and dysregulated proteolysis. Human pathological and clinical studies have aimed to identify select microRNA which may serve as biomarkers of disease and their progression, which are discussed within this review. In addition, I provide comprehensive coverage of in vivo investigations elucidating the modulation of distinct microRNA on the pathophysiology of atherosclerosis, abdominal aortic aneurysms, restenosis, and pulmonary arterial hypertension. Collectively, clinical and animal studies have begun to unravel the complex and often diverse effects microRNAs and their targets impart during the development of cardiovascular diseases and revealed promising therapeutic strategies through which modulation of microRNA function may be applied clinically.

## Graphical abstract

**Figure:**
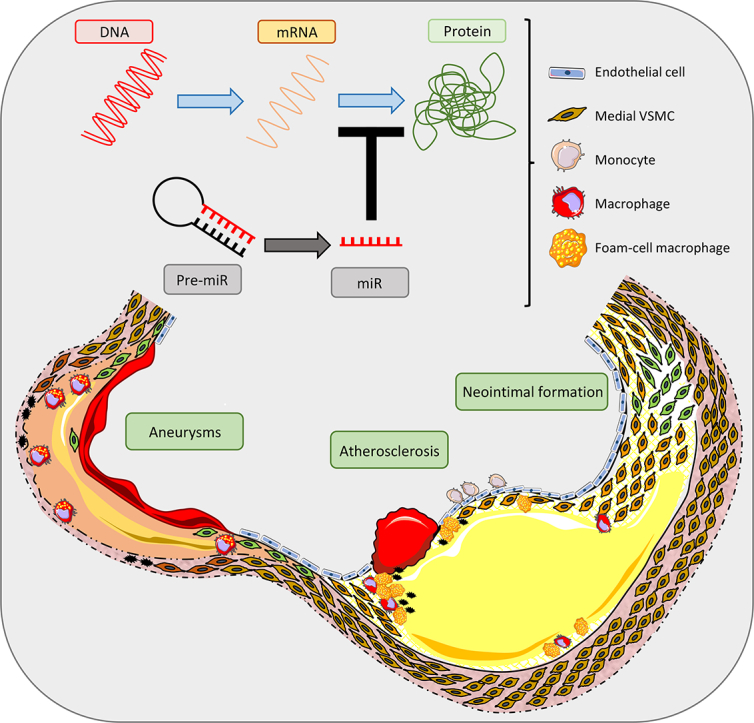

## 1 Introduction

Collectively, the varying forms of cardiovascular disease (CVD) underlie more deaths worldwide than any other illnesses. The underlying process which drives most cardiovascular pathologies is atherosclerosis, a chronic inflammatory disease of the arterial wall involving insudation and retention of lipoproteins at sites of disturbed flow and accompanying dysfunctional endothelium [1]. Advanced coronary artery plaques which give rise to angina and myocardial infarction, are characterised by a lipid-rich/necrotic core associated with focal accumulations of inflammatory cells, particularly lipid-filled macrophages termed foam cells, which is protected from the flowing blood by a vascular smooth muscle cell (VSMC)-rich fibrous cap [2]. Ensuing rupture of an advanced plaque is considered the most common cause of thrombosis and associated clinical events and is attributed to gradual thinning of the thrombo-protective fibrous cap through loss of VSMCs alongside accumulation of highly proteolytic macrophages which can degrade numerous extracellular matrix proteins [3]. Plaque erosion, considered to involve loss of endothelial cells over highly stenotic plaques with accompanying occlusive thrombosis, has recently been proposed as an additional precursor of clinical events [4], although consistent and robust evidence of this phenomenon is still required [3].

Meta-analysis studies have shown patients with abdominal aortic aneurysms (AAA) frequently harbour atherosclerosis [5]. There are also numerous risk factors that are common to the pathogenesis of both pathologies including smoking, hypertension, obesity and age [5]. Genetic risk factors are also shared between AAA and atherosclerosis and a sequence variant on chromosome 9p21 is associated with atherosclerosis and aneurysms [6]. Moreover, intimal atherosclerosis is commonly present in AAA lesions [7], although the composition is different compared to coronary and carotid plaques, and medial elastin fragmentation is more prevalent [5]. Consequently, AAA is considered a form of atherosclerosis with subtle differences in aetiology to those observed in nascent atherosclerosis and is regularly referred to as ‘atherosclerotic aneurysm’ [5,8,9]. Pathological observations suggest that loss of VSMCs, extracellular matrix remodelling in unison with medial and adventitial inflammation drive AAA formation and progression, particularly the transition of small ‘silent’ aortic dilatations to large clinically relevant AAAs [9].

Current clinical intervention strategies to alleviate the consequences of atherosclerotic plaque rupture within coronary arteries includes intravascular stent deployment or coronary artery bypass grafting. However, both interventions result in vascular injury and are associated with recurring clinical presentation requiring reintervention, due to a process known as restenosis. Restenosis involves excessive medial VSMC proliferation and accompanying migration into the intimal portion of the stented artery or bypass graft (usually saphenous vein), resulting in neointimal formation. The newly formed neointima serves as a soil bed for accelerated atherosclerotic plaque formation, commonly termed neoatherosclerosis [10]. Uncontrolled VSMC growth and consequent neointimal formation is also a characteristic observed in many forms of pulmonary arterial hypertension (PAH) [11]. Accordingly, there are numerous mechanistic pathways common between the pathological processes underlying restenosis within coronary arteries after clinical intervention and lesion formation within the arterial tree of the lungs during PAH.

MicroRNAs (miRNAs, miRs) are small noncoding RNA molecules of approximately 18–22 nucleotides in length which can post-transcriptionally regulate gene expression through inhibiting translation or promoting degradation of the target messenger (m)RNA. They are transcribed by polymerase II within the nucleus and are initially produced as primary miRs (pri-miRs). Processing of pri-miRs into their smaller precursor forms (pre-miRs) by RNAse III Drosha is necessary before they can be exported into the cytoplasm. Within the cytoplasmic compartment, pre-miRs are eventually processed into mature and biologically functional microRNA through the action of Dicer, which is another RNAse III family member. Mature microRNA can target and bind the 3′ untranslated regions (3′-UTR) of mRNA and consequently modulate their expression. It has been predicted that microRNAs may modulate up to 90% of mammalian genes and therefore play fundamental roles in regulating cellular function [12]. There is an obvious discrepancy between the number of identified mature microRNA and potential target genes, which is due to the ability of a single microRNA to bind and regulate many differing target mRNA, although there is some evidence that the targets may reside within the same functional networks. There are also more mature microRNAs than precursor forms, owing to the hairpin structure of precursor microRNA and their subsequent processing into -3p and -5p, which can target complimentary and distinct mRNAs. Accordingly, microRNA have been proposed as fine-tuners of gene and protein expression profiles during pathological conditions and correlative studies have assessed the expression of select microRNA and their predicted targets in human pathological samples and tissues from diseased animal models. Furthermore, approaches to modulate microRNA expression or function have been deployed in animal models to determine direct effects on disease progression, including numerous cardiovascular diseases. Modulatory approaches include over-expressing a select microRNA using a miR mimic or viral construct (such as an adenovirus or lentivirus), or retarding function/expression with an antagomir or a genetically-modified mouse with deletion of a specific microRNA.

In this review, I will outline the current knowledge on tissue and circulating microRNA expression changes identified to be involved in four key cardiovascular diseases; atherosclerosis, abdominal aortic aneurysms, restenosis, and pulmonary arterial hypertension. In addition, the results of in vivo animal studies evaluating the effects of modulating specific microRNAs on these leading cardiovascular pathologies will be discussed.

## 2 Atherosclerosis

### 2.1 Human studies

The development, progression and culminating rupture of atherosclerotic plaques underlies most cardiovascular related deaths [13]. It is now widely accepted that atherosclerosis is a chronic inflammatory disease which forms at specific sites within the arterial wall, predilected through haemodynamic changes in blood flow initiating endothelial cell injury and the retention of lipoproteins within an adaptive intimal thickening that develops at such sites [13,14]. Histopathological studies of human atherosclerotic plaques have elucidated that lesion progression and increasing susceptibility to rupture are characterised by monocyte/macrophage infiltration and accumulation, their transformation into foam cells, lipid/necrotic core expansion, a reduction in VSMC number, and decreased collagen content [2]. The recent emergence of microRNAs as key regulators of cellular function and behaviour, such as cell adhesion, invasion and proliferation, lipid uptake and efflux, polarisation, and the release of inflammatory mediators and proteases, has revealed novel mechanistic understanding into their potential role in atherosclerosis, and illuminated them as potential therapeutic targets in conjunction with their identification within the circulation as predictive biomarkers of disease progression.

As previously alluded to, adaptive and pathological intimal thickenings are considered the earliest forms of atherosclerosis within human coronary arteries [15], and a microarray analysis comparing healthy coronary arteries and those harbouring such early lesions revealed that the expression of miR-29, miR-100, miR-155, miR-199, miR-221, miR-363, miR-497, and miR-508 were up-regulated whereas miR-490, miR-1273, and miR-1284 expression were down-regulated [16]. The microRNA expression profile was also examined between non-diseased thoracic arteries and atherosclerotic plaques retrieved from aortic, carotid and femoral arteries, and revealed miR-21, miR-34, miR-146 and miR-210 to be significantly up-regulated in atherosclerotic arteries [17]. A similar study comparing carotid artery atherosclerotic plaques and healthy mammary artery established miR-520 and miR-105 as down-regulated and miR-15, miR-26, miR-30, miR-98, miR-125, miR-152, miR-181, miR-185, and miR-422 as up-regulated in atherosclerotic plaques [18]. Finally, a focussed array of microRNA expression between asymptomatic and symptomatic carotid artery plaques, deemed stable and unstable respectively, revealed expression of miR-100, miR-127, miR-133 and miR-145 were significantly elevated in symptomatic carotid plaques [19]. Assessment of coronary atherosclerotic plaques revealed that miR-181 expression is increased, and miR-24 levels decreased in lesions classified as unstable when compared to stable plaques [20,21].

Fluctuations in circulating microRNAs may also serve as a guide to disease stage and progression. To this end, Leistner and colleagues assessed the correlation of circulating miRs with coronary atherosclerotic plaque burden (assessed by Optical Coherence Tomography) [22]. This study implied that circulating levels of miR-29, miR-126, miR-145, and miR-155 positively correlate with the presence of rupture-prone thin-capped fibroatheroma (TCFA) [22]. A further study deploying comparisons between healthy control subjects and patients with existing coronary artery disease (as defined by previous clinically history but now deemed clinically stable [23]), demonstrated that circulating miR-155, miR-145 and let-7c were significantly decreased in patients with coronary artery disease [23]. Similar studies have shown that whole blood levels of miR-17, miR-19, miR-29, miR-30, miR-92, miR-126, miR-145, miR-150, miR-155, miR-181, miR-222, miR342, miR-378, and miR-484 are decreased in patients with angiographically defined stable coronary artery disease [24,25]. With regards to delineating between stable and unstable coronary artery disease, miR-155 plasma levels were decreased in patients with either unstable angina, acute myocardial infarction or multi-vessel disease, compared to patients with stable or limited disease [26]. A similar approach revealed elevated plasma levels of miR-1, miR-122, miR-126, miR-133, miR-199, miR-433 and miR-485 classified patients with angina, whereas increased miR-337 levels were distinct for patients with stable angina and heightened miR-145 was limited to individuals with unstable angina [27]. Finally, circulating levels of miR-132, miR-150, and miR-186 displayed the greatest discriminatory power for the diagnosis of unstable angina, when compared to patients with non-coronary chest pain or healthy subjects [28].

In addition to assessing microRNA expression within plasma/serum, researchers have also investigated the biomarker potential of microRNA levels within circulating blood cells, especially peripheral blood mononuclear cells (PBMCs), as specific profiles may serve as indicators or predictors of subclinical atherosclerosis and acute coronary syndromes. Evaluation of microRNA expression in peripheral blood cell samples from patients who had suffered an acute myocardial infarction compared to healthy individuals, identified 121 significantly dysregulated microRNA, with miR-663 providing the best predictive value for the presence of acute myocardial infarction [29]. However, due to the study population a proportion of these microRNA (such as miR-145 and miR-30) may be indicators of myocardial damage rather than atherosclerotic plaque disruption [29]. Reduced levels of miR-155 in PBMCs were negatively associated with angiogram-defined coronary artery atherosclerosis and proatherogenic risk factors including age, hypertension, LDL cholesterol levels, and smoking [26]. Assessment of PBMCs from patients carrying a single nucleotide polymorphism in miR-146 (which results in increased expression of the mature form), demonstrated a positive association with coronary artery disease risk in a Chinese cohort [30]. Supportingly, levels of miR-146 are increased in PBMCs of coronary artery disease patients compared to healthy subjects and serves as a predictor of future cardiac events [31]. Comparison of the microRNA signature between patients with coronary artery disease (defined as exhibiting stable or unstable angina) revealed that the expression of miR-135a was increased and miR-147 decreased in PBMCs from coronary artery disease patients, suggesting the miR-135a/miR147 ratio within PBMCs may serve as a predictive tool for atherosclerotic disease risk [32]. Finally, analysis of dysregulated microRNA within CD14 positive monocytes of obese patients revealed down-regulation of several members of the miR-181 family, although only reduced levels of miR-181a was associated with obesity, metabolic syndrome, and angiography-identified coronary artery disease [33].

Collectively, the above findings imply that specific microRNAs detected within the circulation (such as in the plasma, exosomes/microparticles, or within monocytes) may associate with subclinical atherosclerosis. However, patient baseline characteristics, medical treatments, and the presence of contraindicative diseases should be taken into consideration when extrapolating data from profiling studies.

### 2.2 Animal studies

Microarray studies have also been performed in mouse models of atherosclerosis to ascertain microRNAs associated with plaque progression. Using a carotid artery double ligation approach in high fat-fed apolipoprotein E (Apoe) knockout (KO) mice to assess comparisons between plaques deemed unstable and stable or non-diseased, Chen and colleagues identified miR-138, miR-142, miR-322, miR-335, and miR-450 as microRNA up-regulated in advanced plaques with evidence of intraplaque haemorrhage [34]. To directly determine the contributory roles of select microRNA to the development and progression of atherosclerosis, researchers have relied on several complimentary approaches. This primarily involves the use of genetically modified mice which harbour global deletion of Apoe or low density lipoprotein receptor (Ldlr), which renders them hypercholesterolaemic on consumption of a high-fat diet and consequently precipitates atherogenesis at distinct locations within the arterial tree including the aortic root/sinus, carotid and brachiocephalic arteries [35]. Pharmacologically, two main strategies are employed to modulate the activity of individual microRNA in vivo; (1) Restoration or over-expression of an individual microRNA using either synthetic double-stranded RNA molecules (commonly termed mimics or agomirs), or viral expression constructs; and (2) inhibition of microRNA activity through use of chemically modified anti-miR oligonucleotides (commonly termed antagomirs). To date, there have been more than 30 publications assessing microRNA modulation in mouse models of atherosclerosis, the salient findings of which are discussed below (and summarised in Table 1).

**Table 1:** Results of in vivo animal studies evaluating the effects of modulating select microRNA on atherosclerosis.

miRNA(s)	Role	Experimental model – method of microRNA modulation	Cellular origin	Target mRNA	References
miR-let7g	Beneficial	Apoe KO mouse + HFD model – miR mimic	VSMC	LOX1	[36]
miR-19	Detrimental	Apoe KO mouse + HFD model – miR mimic or antagomir	Mac	ABCA1	[37]
miR-21	Beneficial	Ldlr KO mouse + HFD model – miR knockout	Mac	MAP2K3	[38]
miR-24	Beneficial	Apoe KO mouse + HFD model – miR antagomir	Mac	MMP14	[20]
miR-30	Beneficial	Apoe KO mouse + HFD model – miR lentiviral over-expression or miR lentiviral inhibition	Hepatocyte	MTP	[39]
	Beneficial	Apoe KO mouse + HFD model – miR mimic	Hepatocyte		[40]
miR-33	Detrimental	Reversa mouse model ± streptozotocin – miR antagomir	Mono/mac	ABCA1	[48]
	Detrimental/No effect	Apoe KO mouse + HFD model ± bone-marrow transplantation – miR knockout	Mono/mac	ABCG1	[43]
	No effect	Ldlr KO mouse + HFD model – miR antagomir	Mono/mac		[49]
	Detrimental	Ldlr KO mouse + HFD model – miR antagomir	Mono/mac		[46] [47]
	Detrimental	Ldlr KO mouse + HFD model – miR antagomir	Mono/mac		[44]
	No effect/Detrimental	Ldlr KO mouse + HFD model ± bone-marrow transplantation – miR knockout	Mono/mac		[42]
	Detrimental	Ldlr KO mouse + HFD model – miR antagomir	Mac/Hepat		[45]
	Detrimental	Ldlr KO mouse + HFD model – miR antagomir	Hepatocyte		
miR-92	Detrimental	Ldlr KO mouse + HFD model – miR antagomir	EC	SOCS5	[50]
miR-126	Beneficial	Apoe KO mouse + HFD model – miR knockout or mimic	EC	DLK1	[51]
miR-145	Beneficial	Apoe KO mouse + HFD model – miR lentiviral SMC-specific over-expression	EC/VSMC	???	[53]
	Detrimental	Apoe KO mouse + HFD model – miR knockout	VSMC/Mac	ABCA1/SCARB1	[52]
miR-146	Beneficial/Detrimental	Ldlr KO mouse + HFD model ± bone-marrow transplantation – miR knockout or miR antagomir	Mono/mac/EC	SORT	[56]
	Beneficial	Double Apoe:Ldlr KO mouse + HFD model & Ldlr KO mouse +HFD model – miR mimic	Mono/mac	???	[57]
miR-155	Beneficial	Ldlr KO mouse + HFD model ± bone-marrow transplantation – miR knockout	Mono/mac	???	[60]
	Detrimental	Apoe KO mouse + HFD model ± bone-marrow transplantation – miR knockout	Mono/mac	???	[58]
	Detrimental	Apoe KO mouse + HFD model ± bone-marrow transplantation – miR knockout	Mono/mac	BCL6	[59]
miR-181	Detrimental	Apoe KO mouse + HFD model & Ldlr KO mouse +HFD model – miR antagomir	Mac/VSMC	TIMP3/ELN	[21]
	Beneficial	Apoe KO mouse + HFD model – miR mimic	Mac	NOTCH1	[62]
	Beneficial	Apoe KO mouse + HFD model – miR mimic	EC	KPNA4	[61]
miR-182	Detrimental	Apoe KO mouse + HFD model – miR mimic or antagomir	Mac	HDAC9	[65]
miR-223	Beneficial	Apoe KO mouse + HFD model – miR knockout or antagomir	Mono/VSMC	IGF1R	[66]
miR-302	Detrimental	Ldlr KO mouse + HFD model – miR antagomir	Mac/Hepat	ABCA1	[67]
miR-320	Detrimental	Apoe KO mouse + HFD model – miR mimic or antagomir	EC	SRF	[68]
miR-590	Beneficial	Apoe KO mouse + HFD model – miR mimic or antagomir	Mac	LPL	[70]
miR-712 (miR-205)	Detrimental	Apoe KO mouse + HFD model ± carotid ligation – miR mimic or antagomir	EC	TIMP3	[71]

#### 2.2.1 miR-let-7g

Using such approaches, a protective role for miR-let-7g has been proposed as intravenous delivery of a miR-let-7g specific mimic attenuated the development of atherosclerotic lesions within the aorta of hypercholesterolaemic Apoe KO mice [36]. Further in vitro studies demonstrated that miR-let-7g can directly target and suppress protein expression of LOX-1 in aortic VSMCs, and consequently repress their migratory and proliferative capacity in response to oxLDL, in line with the effects observed in vivo [36].

#### 2.2.2 miR-19

Gain and loss-of-function studies in Apoe KO mice suggested that miR-19 supports atherogenesis through promotion of macrophage foam cell formation, as miR-19 directly regulates macrophage expression of ABCA1, a key regulator of macrophage cholesterol efflux [37]. Accordingly, systemic administration of a miR-19 mimic to Apoe KO mice reduced plasma HDL levels and concomitantly elevated LDL concentration [37].

#### 2.2.3 miR-21

Adoptive transfer of miR-21 deficient hematopoietic cells worsened atherosclerosis within the aortic arch and thoracic aorta of Ldlr KO mice [38]. The protective effect of miR-21 in atherosclerosis was attributed to promoting macrophage survival and their phagocytic capacity alongside preservation of ABCG1 expression, a positive regulator of cholesterol efflux which is negatively regulated by the miR-21 target MAP2K3 (also known as MKK3) [38]. However, miR-21 expression is elevated in human plaques when compared to non-diseased arteries, although this may simply reflect the presence of macrophages [17].

#### 2.2.4 miR-24

Findings from human macrophages and coronary plaques have shown that down-regulation of miR-24 promotes macrophage invasion through increased matrix metalloproteinase (MMP)-14 activity and associates with plaque instability [20]. Indeed, a proof-of-principle study in atherosclerotic Apoe KO mice demonstrated that miR-24 inhibition accelerated atherosclerosis in brachiocephalic arteries which was linked with heightened intra-plaque macrophage MMP-14 expression [21].

#### 2.2.5 *miR-30*

It has been recently shown that miR-30 levels within the liver orchestrates packaging and secretion of apoB-containing lipoproteins such as VLDL and LDL, by regulating expression of the microsomal triglyceride transfer protein (MTTP) [39]. Pharmacologically, either lentiviral over-expression of miR-30 or liver-directed delivery of a miR-30 mimic mitigated hypercholesterolaemia and aortic atherosclerosis in Apoe KO mice, without inducing hepatosteatosis (an undesirable side effect of conventional MTTP inhibitors) by diminishing hepatic lipid synthesis [39,40].

#### 2.2.6 *miR-33*

Despite a lack of evidence linking alterations in miR-33 expression to human atherosclerosis, numerous studies employing antagomir inhibition or gene deletion approaches have been undertaken in mouse models of atherosclerosis to ascertain the contributory role of miR-33. Most of the studies, conducted exclusively in Ldlr KO mice, suggest a deleterious role for miR-33 on the development and progression of aortic atherosclerosis. It is evident that miR-33 exerts a key regulatory role in lipid homeostasis, including key pathways for controlling cholesterol and fatty acid equilibrium [41,42]. Moreover, whole mouse deletion of miR-33 increased plasma levels of HDL cholesterol and reduced aortic root atherosclerotic plaque size in Apoe KO mice [43]. However, hematopoietic cell-restricted deletion of miR-33 did not affect HDL-cholesterol levels or plaque size, although lipid accumulation within lesions was reduced, attributed to enhanced cholesterol efflux from foam cell macrophages through restoration of ABCA1 and ABCG1 expression [43]. Conversely, whole body deletion of miR-33 in Ldlr KO mice did not influence the progression of aortic atherosclerosis despite inducing marked dyslipidaemia, whereas hematopoietic-specific loss of miR-33 hampered plaque development but did not impact on circulating lipid levels [44]. Moreover, whole mouse deletion of miR-33 was also associated with the development of obesity and insulin resistance, while such deleterious effects were not reported in the hematopoietic-specific miR-33 KO animals, and the beneficial effects on atherogenesis attributed to enhanced cholesterol efflux from plaque macrophages, limiting lipid accumulation and further inflammatory cell recruitment [44]. As such, therapeutically it would be advantageous to target macrophage specific miR-33. Nonetheless, several studies have demonstrated that systemic miR-33 inhibition can attenuate atherosclerotic plaque development and progression in Ldlr KO mice [42,[45], [46], [47]] or diabetic REVERSA mice [48], with no or varying effects on plasma LDL-cholesterol and HDL-cholesterol levels. However, using a similar approach, Marquart and colleagues observed no preventative effect of long-term (14 weeks) miR-33 inhibition on aortic atherosclerosis in high-fat fed Ldlr KO mice [49]. Despite the reported favourable effects of miR-33 antagonism on circulating levels of HDL-cholesterol and atherosclerosis, worrying elevations in circulating triglyceride levels alongside development of hepatosteatosis have been described [43,49]. Taken together, these above findings suggest that to utilise miR-33 therapeutically, a cell-specific delivery approach will be necessary to ensure selective targeting of macrophages.

#### 2.2.7 *miR-92*

The up-regulated expression of miR-92 specifically in endothelial cells has been associated with arterial sites deemed atherosclerosis-prone due to haemodynamic changes and LDL modification, in humans and Ldlr KO mice [50]. Accordingly, down-regulation of miR-92 achieved through delivery of a specific inhibitor to hypercholesterolaemic Ldlr KO mice, retarded plaque development and favourably altered lesion composition, ascribed to restored endothelial cell expression of the negative regulator of cytokine signalling, SOCS5 [50].

#### 2.2.8 *miR-126*

Haemodynamic alterations also modulate endothelial cell expression of miR-126, as altered shear stress present at atherosclerosis predilection sites suppresses endothelial miR-126 levels and reduces their proliferative capacity due to perturbation of Notch signalling in a DLK1-dependent manner [51]. Using an endothelial-denudation approach in Apoe KO mice, systemic delivery of a miR-126 mimic decreased plaque size, while lesion area was increased in miR-126 antagomir-treated animals [51]. Further supporting an advantageous role for miR-126, Apoe KO mice also deficient for miR-126 displayed accelerated aortic atherogenesis at both predilection and non-predilection sites [51].

#### 2.2.9 *miR-145*

Evidence from studies evaluating microRNA expression in plasma and atherosclerotic plaques of patients with advanced atherosclerosis have provided a strong association between elevated miR-145 levels and disease progression [19,22]. In line with these findings, Ldlr KO mice which are also deficient for miR-143 and miR-145 were protected from the progression of aortic atherosclerosis [52]. Intriguingly, it was proposed that VSMC miR-145 can be transferred to macrophages in response to atherogenic stimuli, inducing ABCA1 down-regulation, reduced cholesterol efflux, and enhanced foam cell formation [52]. Conversely, miR-145 expression has also been shown to be attenuated in Apoe KO mouse and human atherosclerosis, although it must be noted that human plaques were not stratified by histological phenotype or clinical characteristics, and were compared to plaque-free arteries [53]. Although further support for a beneficial effect of miR-145 comes from results demonstrating that circulating levels of miR-145 are reduced in patients with coronary artery disease compared to control subjects [24]. Furthermore, VSMC-specific over-expression of miR-145 retarded plaque progression in Apoe KO mice which was associated with directing VSMCs towards a contractile phenotype [53]. In addition, it has been shown that under athero-protective stimuli such as laminar shear stress, endothelial cells release extracellular vesicles rich in miR-143 and miR-145 which are transported to VSMCs to promote a contractile phenotype [54]. The paradoxical findings reported above demonstrate the need for further studies to elucidate the therapeutic and diagnostic potential of miR-145.

#### 2.2.10 *miR-146*

The expression of miR-146 is elevated within human atherosclerotic plaques of the aorta and femoral artery [17]. In addition, a single nucleotide polymorphism in the *miR146a* gene which influences miR-146a expression may serve as a predictor for susceptibility to coronary artery disease [55]. Mice deficient for both Ldlr and miR-146 display suppressed atherosclerosis within the aortic arch which was associated with reduced circulating LDL cholesterol levels, which alongside the human findings supports a deleterious role for this microRNA [56]. Complimentary bone-marrow transplantation experiments showed similar findings in Ldlr deficient mice receiving miR-146 knockout bone marrow, suggesting monocyte/macrophage-derived miR-146 is the dominant effector of atherogenesis in both models, through targeting of SORT1 and associated regulation of circulating LDL levels [56]. In opposition, a protective effect for miR-146 has been proposed as administration of a miR-146 mimic to either Apoe and Ldlr double knockout mice or Ldlr deficient only mice retards aortic root atherosclerosis [57]. Further investigations demonstrated that Apoe regulated macrophage miR-146 levels to dampen NFκβ-driven pro-inflammatory responses [57]. As such, further studies are required to elucidate the biomarker potential and contributory role for miR-146 to plaque progression.

#### 2.2.11 *miR-155*

The expression of miR-155 is elevated in both experimental mouse and human plaques [58,59], implying a progressive role for this microRNA in atherosclerosis. Indeed, loss- and gain-of-function in vitro studies have demonstrated that macrophage miR-155 expression is associated with foam cell formation [58] and promoting a pro-inflammatory macrophage phenotype, in part through direct repression of BCL6 and therefore promoting NFκβ-activity [59]. In vivo, global [58] or bone-marrow restricted deletion [58,59] of miR-155 in Apoe knockout mice suppressed aortic root atherogenesis, which could be reversed through localised BCL6 silencing [59]. Conversely, bone marrow transplantation studies conducted in Ldlr deficient mice revealed hematopoietic miR-155 deficiency accelerated atherogenesis and therefore intimates that miR-155 exerts anti-inflammatory and athero-protective effects particularly during hypercholesterolaemia and is associated with suppression of circulating inflammatory monocyte numbers and restoration of IL-10 production [60]. It is possible the divergent effects reported above are in part due to the different mouse models of atherosclerosis deployed. Indeed, the levels of hypercholesterolaemia observed in Apoe and Ldlr deficient mice differ markedly and macrophage foam cell formation dynamically regulates miR-155 expression [60].

#### 2.2.12 *miR-181*

The miR-181 family members are differentially expressed between human classical and non-classical monocyte subsets, and elevated in human carotid plaques compared to non-diseased arteries [18]. Furthermore, miR-181b expression is increased in unstable coronary plaques compared to their stable counterparts, predominantly by pro-inflammatory foam cell macrophages [21]. Accordingly, in vivo administration of a miR-181b inhibitor retarded both the development and progression of atherosclerotic plaques in Apoe knockout mice and Ldlr deficient animals [21]. It was determined that miR-181b negatively regulates TIMP-3 expression in macrophages and ELN levels in vascular smooth muscle cells [21]. Conversely, two studies revealed circulating miR-181b levels are decreased in patients with coronary artery disease [61] or suffering from acute stroke [62]. Furthermore, utilising systemic delivery of miR-181b mimics (also termed agomirs) to Apoe knockout mice, elevating circulating exogenous miR-181b levels retarded atherosclerotic lesion formation [61,62]. The beneficial effects of miR-181b were attributed to suppression of endothelial cell IPOA3 expression and associated dampening of NFκβ-activity [61], and promoting anti-inflammatory macrophage polarisation through repressed NOTCH1 expression and downstream signalling [62]. The discrepancies between the above studies may reflect the differing efficacies of miR mimics and locked nucleic acid (LNA)-modified miR inhibitors to target the atherosclerotic plaque; as LNA-miR inhibitors are more potent and they have the potential to affect all cells within the lesion [63].

#### 2.2.13 *miR-182*

Plasma concentrations of miR-182 are elevated in coronary artery disease patients compared to healthy controls, implying miR-182 may serve as a biomarker of atherosclerosis progression [64]. Supporting a deleterious role for miR-182, systemic delivery of an agomir (mimic) to high fat fed Apoe knockout mice accelerated aortic atherosclerosis compared to control animals, while administration of an antagomir suppressed plaque development [65]. Mechanistic studies revealed miR-182 targets and down-regulates the histone deacetylase HDAC9 in macrophages, resulting in increased lipoprotein lipase (LPL) expression which in turn facilitates lipid accumulation and subsequent pro-inflammatory foam cell macrophage formation [65].

#### 2.2.14 *miR-223*

Levels of miR-223 are elevated within the plasma and diseased vessels of atherosclerotic Apoe knockout mice and patients [66]. Leukocytes and platelets serve as the major sources of miR-223, which can be transported into the vessel wall via microparticles and be subsequently taken up by vascular smooth muscle cells to down-regulate IGF-1R expression and retard cell growth [66]. Indeed, delivery of a miR-223 inhibitor reduced aortic atherosclerotic area in Apoe knockout mice [66]. However, this may be to the detriment of plaque stability as miR-223 deficient mice display increased neointimal formation than wild-type mice after carotid artery ligation injury [66]. Therefore, while miR-223 inhibition may have therapeutic potential for limiting atherogenesis and restenosis, it may precipitate plaque rupture and thus negate its systemic deployment in atherosclerotic patients.

#### 2.2.15 *miR-302*

A genome-wide screening study evaluating dysregulated microRNA in macrophages with and without exposure to modified LDL, revealed miR-302 was inversely correlated with cholesterol efflux [67]. Mechanistic studies identified ABCA1 as a direct target of miR-302 in macrophages and within the liver, therefore forced expression of miR-302 diminishes cholesterol efflux and fosters both foam cell macrophage formation and aberrant hepatic cholesterol clearance [67]. As such, treatment of Ldlr knockout mice with a miR-302 inhibitor increased circulating HDL levels and reduced aortic atherosclerosis progression, including perturbed necrotic core size even in the face of heightened macrophage accumulation [67].

#### 2.2.16 *miR-320*

Patients with coronary artery disease exhibit markedly increased circulating levels of miR-320 compared to healthy controls [68]. Supporting a deleterious role for miR-320, systemic plasmid-derived over-expression of miR-320 promoted atherogenesis in Apoe deficient mice, which was associated with induction of endothelial dysfunction, while miR-320 anti-sense delivery attenuated atherosclerosis [68]. Confirmatory in vitro findings demonstrated that miR-320 directly targets and decreases endothelial cell expression of SRF, retarding cellular proliferation and promoting their susceptibility to apoptosis [68].

#### 2.2.17 *miR-590*

In contrast to miR-182 which augments LPL expression through regulation of HDAC9, miR-590 directly targets and represses macrophage LPL levels, thus retarding the formation of pro-inflammatory foam cell macrophages [69,70]. Accordingly, in high fat-fed Apoe knockout mice, systemic administration of a miR-590 agomir (mimic) prevented the progression of aortic atherosclerosis, while delivery of a miR-590 antagomir increased atherogenesis [70]. As expected, intraplaque macrophage LPL expression was modulated in both experiments, and reciprocal changes in circulating LDL-cholesterol levels were also observed [70].

#### 2.2.18 *miR-712*

Assessment of microRNAs dysregulated by athero-prone disturbed flow on mouse endothelial cells in vitro and in vivo identified miR-712 as a mechanosensitive microRNA [71]. Mechanistic findings demonstrated miR-712 down-regulates endothelial cell TIMP-3 expression, promoting aberrant proteolysis and promoting endothelial inflammation and permeability [71]. As such, knockdown of miR-712 through systemic delivery of miR-712 antagomir prevented atherogenesis in Apoe deficient mice subjected to partial left carotid ligation and was associated with restored vessel wall TIMP-3 expression [71]. Validative studies in human endothelial cells identified miR-205 as a homologue of murine miR-712 and confirmed miR-205 represses endothelial cell TIMP-3 expression in a mechanosensitive manner [71].

**Fig. 1:**
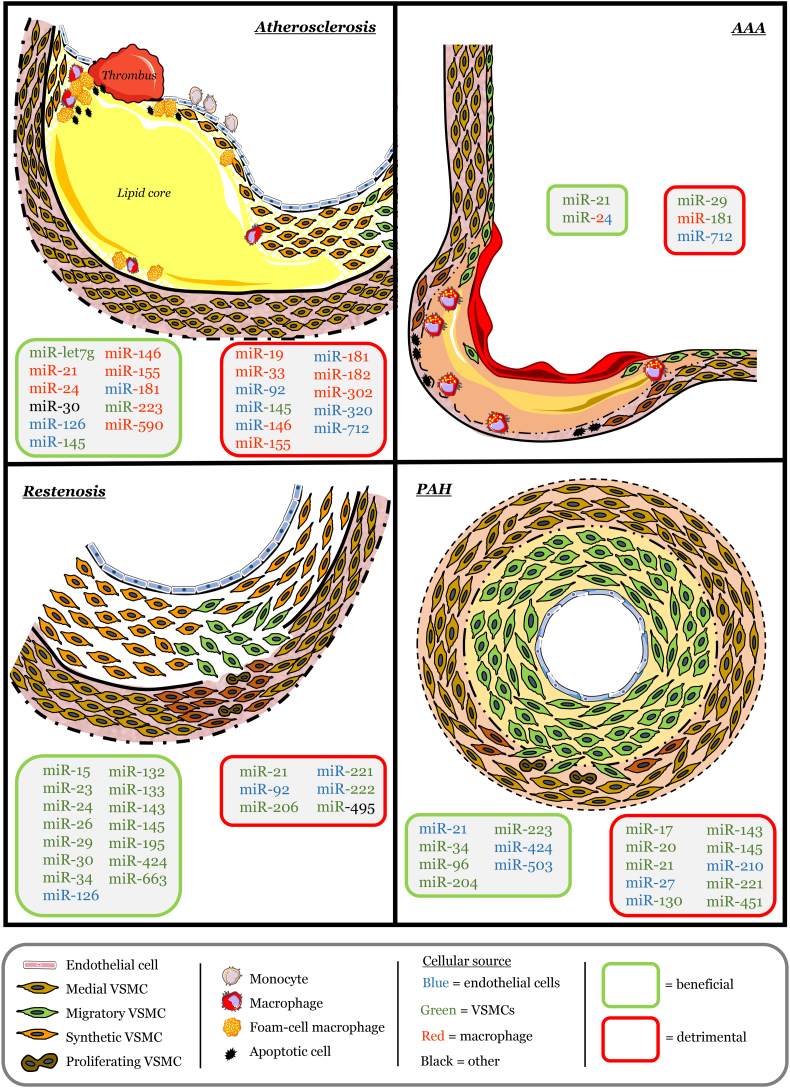
MicroRNA identified in animal studies to exert beneficial or detrimental effects on cardiovascular diseases.

Through studies conducted in isolated cells and animal models, alongside human pathological and clinical findings, distinct microRNAs have been identified and proposed to play key roles in the development, progression, and disruption of atherosclerotic plaques. Seminal studies utilising animal models that permit modulation of individual microRNAs has permitted the identification of specific beneficial and detrimental roles select microRNA exert on differing target RNA, and the ensuing significance to atherosclerosis (summarised in Fig. 1). Collectively, this large body of work has identified certain microRNA which may serve as therapeutic targets to prevent disease progression, affecting processes such as cell turnover, proteolysis, and lipid metabolism. However, the importance of identifying the specific cell types expressing select microRNA is essential when attempting to attribute causality to multifactorial diseases such as atherosclerosis, especially when conclusions are derived from whole tissue analysis [72]. For example, although miR-145 levels are increased in patients with identified atherosclerosis, this may reflect ongoing processes within VSMCs to provide stability to plaques through maintenance of the fibrous cap. Indeed, the VSMC-specific miR-145 over-expression study demonstrating direct effects on VSMC phenotypic modulation [53] supports such a proposition when taken alongside underpinning studies establishing a fundamental role for miR-145 (and miR-143) in regulating VSMC function and plasticity [[72], [73], [74]].

## 3 Abdominal aortic aneurysms

### 3.1 Human studies

The majority of abdominal aortic aneurysms (AAAs) are clinically silent or asymptomatic until rupture, and for this reason they are difficult to detect, and ruptures are linked to mortality in 85–90% of cases [75]. Pharmacological therapies alongside recurrent imaging are utilised for small aneurysms (less than 5.5 cm in diameter) to limit their progression [76]. However, when aorta dilatation exceeds 5.5 cm, elective surgical repair is adopted despite mortality percentages remaining high after surgery [75]. Studies have shown that patients with AAAs frequently have atherosclerosis [77], and are regularly referred to as “atherosclerotic aneurysms” [5,78]. Similar to coronary atherosclerosis, extracellular matrix remodelling in unity with the accrual of inflammatory cell infiltrate, especially macrophages, at both the adventitial and medial aspects is a prominent feature of human atherosclerotic AAAs [79], and also observed in pre-clinical animal models of AAAs [80]. Accordingly, evidence from both human pathological studies and mouse AAA models have supported a deleterious role for inflammation and MMPs in AAA progression [83].

Array studies alongside focussed RNA analysis of human AAA and normal aortic tissue samples have identified numerous miRNA which are differentially expressed. However, due in part to the heterogeneity of AAA tissues owing to differences in grade, size, inflammatory nature, and location of tissue resection, a limited number of miRNA have been consistently identified. While miR-let7, -15a/b, -21, -29b, -124a, -126, -146a, -155, -181a/b, -205, and -223 have been observed to be up-regulated in AAA tissues, and miR-24, -30c, -133a/b, -204, and 331a down-regulated, only expression of miR-21 and -146a have been confirmed to be increased in AAA compared to healthy control tissues in multiple studies [84,85]. Companion studies have also been performed to determine if circulating miRNA levels can be deployed as biomarkers of AAA progression. Plasma levels of miRs shown to exhibit the largest increase in AAA patients compared to controls, include miR-let-7i, -33a, -191, -331, -411, -455, -652, and -1281 [[86], [87], [88]]. Conversely, miR-let-7e, -10b, -15a/b, -16, -29b, -124a, -126, -146a, -155, -192, -194, -195, -196b, -205, -215 and -223 have all been reported to be decreased in the peripheral blood of AAA patients compared to controls [[86], [87], [88], [89]]. While some of the altered levels in circulating miR levels agree with changes observed in AAA tissues, many studies have shown inverse correlations, for example miR-29b and miR-146a are increased in AAA tissue and decreased with in peripheral blood [85], highlighting the caveats of deploying miRNAs as potential biomarkers of AAA progression. It is clear larger robust studies are required to correlate peripheral blood miRNA levels and associated AAA tissue expression, to elucidate the role of select miRNAs in AAA formation and progression. Further work should also determine the cellular/tissue sources of circulating miRNA, as these may be derived from an array of different depots including circulating immune cells, other cells/organs affected by AAA, or the diseased aortic wall, in order to evaluate if the circulating miRs are causal or a consequence of AAA. Finally, in humans, AAA is associated with atherosclerosis and therefore altered circulating miR levels may reflect the presence of atherosclerosis (within the coronary vasculature for example). However, analysis of plasma miRs between AAA patients and CAD-only individuals revealed that changes in circulating levels of miR-124a, -155, and -223a were AAA-specific [85], and may therefore serve as biomarkers of end-stage AAA.

Although not the focus of this review, the pathophysiology of AAA shares strong similarities to thoracic aortic aneurysms (TAA) and as such many of the miRs identified to play a role in AAA formation and progression have also been suggested to modulate TAAs, discerned through assessment of tissue and circulating miR expression patterns alongside a small number of in vivo mouse studies (as reviewed by [90]). Indeed, a meta-analysis study examining association of changes in miRNAs within AAA and TAA patients revealed a portfolio of miRs dysregulated in both forms of aortic aneurysm, including miR-24, -29, -30, -133, -143, -145, −193, -223, and -933 [91]. In vivo studies have also demonstrated that modulation of specific miRNAs (such as miR-29 and miR-181b) exert similar effects on AAA and TAA formation and progression [21,92]. Another clinically-relevant site of aneurysm formation is within the cerebral artery, and therefore commonly termed intracranial aneurysm, and gives rise to complications including subarachnoid haemorrhage, attributed to increasing inflammation and dysregulated proteolysis [93]. Despite limited research into the contributory roles of microRNA to intracranial aneurysms development and rupture, genome-wide microRNA screening identified 72 upregulated and 85 downregulated microRNA in intracranial aneurysm tissues compared to normal temporal arteries, including miR-99b, miR-340, and miR-493 [94]. Biomarker-associated approaches utilising assessment of plasma has revealed three circulating microRNA which can discriminate intracranial aneurysm patients from controls, miR-let7b, miR-183, and miR-200a [95]. Interestingly, miR-29b has recently been identified as a potential microRNA of therapeutic interest from expression studies in intracranial tissues and mechanistic studies revealing a role for this microRNA in regulating VSMC phenotypic modulation [96].

### 3.2 Animal studies

There are limitations associated with studies involving human AAA tissues, including the use of end stage disease samples, which limits elucidation of the contribution of miRs to AAA development and progression. Furthermore, pathological tissues and associated plasma samples provide guilt by association but do not permit mechanistic evaluation or determination of causal roles of specific miRs to AAA pathogenesis. Accordingly, there is the necessity to use animal models of AAA formation and progression to assess the contributory roles of miRs identified as potential modulators of AAA. The angiotensin II (Ang II)-infused hypercholesterolaemic mouse model and the calcium chloride peri-vascular application model are the most commonly utilised for mechanistic AAA studies [97], and have been deployed to ascertain the contributory roles of select microRNA to aortic aneurysm formation and progression (summarised in Table 2).

**Table 2:** Results of in vivo animal studies evaluating the effects of modulating select microRNA on abdominal aortic aneurysm (AAA) formation.

miRNA(s)	Role	Experimental model – method of microRNA modulation	Cellular origin	Target mRNA	References
miR-21	Beneficial	C57Bl/6 J mouse + elastase-induced AAA – miR mimic or antagomir	VSMC	PTEN?	[98]
		Apoe KO mouse + Ang II-induced AAA – miR mimic or antagomir			
miR-24	Beneficial	C57Bl/6 J mouse + elastase-induced AAA – miR mimic or antagomir	Mac/VSMC/EC	CHI3L1	[99]
		C57Bl/6 J mouse + Ang II-induced AAA – miR mimic or antagomir			
miR-29	Detrimental	C57Bl/6 J aged mouse + Ang II-induced AAA – miR antagomir	VSMC	COL1A1?	[92]
	No effect	Apoe KO mouse + Ang II-induced AAA – miR antagomir		COL3A1?	[92]
	Detrimental	C57Bl/6 J mouse + elastase-induced AAA – miR mimic or antagomir	Fibroblasts	FBN1?	[101]
	Detrimental	Apoe KO mouse + Ang II-induced AAA – miR mimic or antagomir		ELN?	[101]
	Detrimental	Apoe KO mouse + Ang II-induced AAA – miR antagomir	VSMC	MMP2?	[89]
	Detrimental	Fbn1^C1039G/+^ ‘Marfan’ mouse model of AAA – miR antagomir	VSMC	BCL2	[102]
	Detrimental	Fbn1^C1039G/+^ ‘Marfan’ mouse model of AAA – miR antagomir	VSMC		[103]
miR-181	Detrimental	Apoe KO mouse + Ang II-induced AAA – miR antagomir	Mac/VSMC	TIMP3	[21]
		Ldlr KO mouse + Ang II-induced AAA – miR antagomir		ELN	
miR-195	No effect	Apoe KO mouse + Ang II-induced AAA – miR antagomir	VSMC	???	[89]
miR-712 (miR-205)	Detrimental	Apoe KO mouse + Ang II-induced AAA – miR antagomir	EC	TIMP3	[105]
				RECK	

#### 3.2.1 *miR-21*

In agreement with the human data, miR-21 expression is elevated in Ang II-induced mouse AAA tissues [98]. However, miR-21 inhibition through use of an antagomir promoted AAA expansion, attributed to increased phosphatase and tensin homologue (PTEN) protein expression and an associated anti-proliferative effect on VSMCs [98]. Conversely, lentiviral over-expression of miR-21 retarded AAA growth, repressed PTEN expression and promoted survival and proliferation of VSMCs [98]. This study suggests that the observed up-regulation of miRs within AAA tissues does not necessarily translate to them playing a deleterious role but may represent a counterregulatory physiological response. As such, caution should be employed when extrapolating changes in miR expression to pathological guilt.

#### 3.2.2 *miR-24*

AAA retrieved from mouse elastase-infusion or Ang II-induced models exhibited reduced miR-24 levels, in accordance with findings from human AAA tissues [99]. Accordingly, in both models forced increased expression of miR-24 retarded AAA formation while miR-24 inhibition accelerated disease progression, ascribed to miR-24 regulating Chitinase 3-like 1 (Chi311) expression and associated macrophage-driven vascular inflammation [99]. These results are somewhat surprising given that Chi3l1 is commonly used as a marker of the anti-inflammatory ‘M2’ macrophage subset in mice, which have been shown to limit the magnitude and duration of inflammatory responses and promote wound healing [100].

#### 3.2.3 *miR-29*

There are several lines of evidence supporting a deleterious role for miR-29 in AAA formation and progression. Utilising both the Ang II-infusion and elastase-infusion models, enhancing circulating miR-29 levels augmented AAA growth [101], whereas miR-29 inhibition limited AAA expansion [89,101]. Gene and proteomics analysis of mouse AAAs suggested the principal targets of miR-29 included numerous VSMC generated extracellular matrix proteins (Col1a1, Col3a1, Col5a1, and Eln) and matrix-degrading enzymes (MMP-2 and MMP-9) [89,101], which were also validated in human AAA tissues [89]. Interestingly, in a mouse model of Marfan syndrome which contains a mutation in the Fibrillin1 gene and spontaneously develops aneurysms throughout the aorta, miR-29 levels are increased, and miR-29 inhibition prevents aneurysm formation [102] and progression [103].

#### 3.2.4 *miR-181*

Multiple studies have identified that miR-181a/b expression is increased in tissues and plasma from AAA patients [21,87,88] and associates with pro-inflammatory macrophages [21]. However, evidence in mice and supported by in vitro mechanistic studies suggested that miR-181b can suppress vascular inflammation, in part through regulation of NF-κβ signalling in endothelial cells by targeting importin-α3, a protein necessary for NF-κβ translocation [104]. Still, AAA growth and severity in Ang II-infused hypercholesterolaemic Apoe-deficient or Ldlr-deficient mice was reduced through inhibition of miR-181b [21]. Limiting miR-181b expression was associated with increased macrophage TIMP-3 expression and VSMC elastin levels, both important factors for maintaining aneurysm stability [21].

#### 3.2.5 *miR-205*

Expression of miR-712 and its human homologue miR-205 are increased in mouse and human AAA tissues, respectively [105]. Moreover, elevated levels of miR-712/−205 accompanied decreased expression of the endogenous MMP inhibitors TIMP-3 and reversion-inducing cysteine-rich protein with kazal motifs (RECK), and concomitant increased MMP activity in endothelial cells of AAAs [105]. Inhibition of either miR-712 or miR-205 in the Ang II-induced mouse AAA model limited disease formation, restored TIMP3 and RECK expression, reduced MMP activity and diminished elastin fragmentation [105].

Collectively, the numerous mouse and in vitro studies moderating miR expression have afforded a better understanding of their specific roles to AAA formation and progression (summarised in Fig. 1). As expected, the microRNA identified target key molecules involved in either SMC function, inflammation, or proteolysis, three key mechanisms associated with the progression of AAAs in humans. However, it remains to be seen if targeting the aforementioned select miRNAs serves as a feasible therapeutic approach for the treatment of human AAA. Considering the multifactorial nature of AAA pathophysiology, targeting multiple microRNA may provide more efficacious, whilst always considering the potential off-target effects.

## 4 Restenosis

### 4.1 Human studies

The dysregulated accumulation of VSMCs into the intimal region of blood vessels and their associated deposition of extracellular matrix proteins underlies many occlusive cardiovascular diseases, including atherosclerosis, restenosis, and pulmonary arterial hypertension [106]. VSMCs reside within the media of healthy blood vessels in a quiescent and contractile state. However, after vascular injury such as encountered upon stent deployment or coronary artery bypass grafting, VSMCs undergo phenotypic modulation, proliferate and migrate into the intima resulting in a process commonly termed neointimal formation or intimal hyperplasia [107]. Excessive neointimal formation can compromise lumen patency and blood flow restriction directly through restenosis, or indirectly by facilitating accelerated superimposed atherosclerosis [107]. Recent discoveries have proposed a major contributory role for microRNAs to the pathobiological mechanisms underlying restenosis after stent deployment or saphenous vein bypass grafts. Consequently, select microRNAs may serve as novel selective therapeutic targets or circulating biomarkers to treat and diagnose susceptibility to restenosis after arterial or bypass vascular injury.

Due to difficulties in retrieving tissue samples from diseased or failed stented coronary arteries and saphenous vein grafts, there is limited data on microRNA dysregulation during neointimal formation after vascular injury in humans. Some studies have demonstrated the presence of focal accumulations of specific microRNAs within human neointimal formation. Indeed, both failed vein graft tissues and stented coronary arteries with developing neointimal formation showed abundant miR-21 expression [108,109]. There is also limited assessment of the effect of vascular injury on circulating microRNA levels in human. However, patients deemed to display in-stent restenosis as assessed by angiography, exhibited elevated plasma levels of miR-21 while miR-100, -143, and -145 were decreased, when compared to patients which did not develop marked restenosis after stent deployment, or healthy non-diseased controls [110]. Though no significant difference in circulating levels of miR-31, -125b, -130a, -146a, -210 and -221 were detected between the three groups [110]. Several members of the miR-17-92 cluster are upregulated in carotid arteries exhibiting restenosis after angioplasty and stenting when compared to adjacent normal artery, including miR-17, -18a, -19a, -20a, and -92a [111].

### 4.2 Animal studies

Rodent and large animal models of vascular injury, particularly the rat carotid balloon injury, and the mouse and porcine stent models have been used to examine the expression and contributory roles of microRNAs to neointimal formation (summarised in Table 3). Indeed, microarray analysis of rat carotid arteries after angioplasty revealed numerous dysregulated microRNA when compared to uninjured control arteries; miR-21, -146, -214, -221, -222 and -352 were highly upregulated, whereas miR-125a, -125b, -133a, -143, -145, -347, and -365 were significantly downregulated [112,113].

**Table 3:** Results of in vivo animal studies evaluating the effects of modulating select microRNA on neointimal formation/restenosis. Treatments were administered systemically unless otherwise stated.

miRNA(s)	Role	Experimental model – method of microRNA modulation	Cellular origin	Target mRNA	References
miR-1	No effect	Rat carotid artery balloon injury model – miR adenoviral over-expression (local)	VSMC	???	[130]
miR-15a/b	Beneficial	Rat carotid artery balloon injury model – miR adenoviral over-expression (local)	VSMC	YAP	[115]
miR-21	Detrimental	Rat carotid artery balloon injury model – miR antagomir (local)	VSMC	PTEN	[112]
	Detrimental	Mouse stented aorta-interposition graft model – miR knockout		BMPR2	[109]
	Detrimental	Rat stented internal mammary artery balloon injury model – miR antagomir coated stents		BCL2	[114]
	Detrimental	Mouse isogenic vein graft model – miR knockout		PPARG	[108]
miR-23	Beneficial	Rat carotid artery balloon injury model – miR adenoviral over-expression (local)	VSMC	FOXO4	[117]
miR-24	Beneficial	Diabetic rat carotid artery balloon injury model – miR adenoviral over-expression (local)	VSMC	WNT4	[118]
miR-26	Beneficial	Rat autogenous jugular vein graft model – miR lentiviral over-expression (local)	VSMC	MAPK6	[119]
miR-29	Beneficial	Rat carotid artery balloon injury model – miR mimic (local)	VSMC	MCL2 & MMP2	[120]
miR-30	Beneficial	Rat carotid artery balloon injury model – miR lentiviral over-expression (local)	VSMC	CAMK2D	[121]
miR-34	Beneficial	Mouse wire injury femoral artery model – miR mimic (local)	VSMC	NOTCH1	[123]
	Beneficial	Rat carotid artery balloon injury model – miR mimic (local)		SCF	[124]
miR-92	Detrimental	Rat carotid artery balloon injury model & stenting model – miR antagomir	EC	KLF4	[126]
miR-126	Beneficial	Mouse wire injury carotid artery model – miR mimic and antagomir (EC-derived microparticles)	EC	LRP6	[127]
miR-132	Beneficial	Rat carotid artery balloon injury model – miR mimic (local)	VSMC	LRRFIP1	[129]
miR-133	Beneficial	Rat carotid artery balloon injury model – miR adenoviral over-expression (local)	VSMC	SP1 & MSN	[130]
miR-143	Beneficial	Rat carotid artery balloon injury model – miR mimic (local)	VSMC	KLF5	[131]
miR-145	Beneficial	Rat carotid artery balloon injury model – miR mimic (local)	VSMC	KLF5	[131]
	Beneficial	Rat carotid artery balloon injury model – miR adenoviral over-expression (local)			[132]
miR-195	Beneficial	Rat carotid artery balloon injury model – miR adenoviral over-expression (local)	VSMC	CDC42 & CCND1	[133]
miR-206	Detrimental	Rat carotid artery balloon injury model – miR lentiviral knockdown (local)	VSMC	ZFP580	[134]
miR-208	No effect	Rat carotid artery balloon injury model – miR mimic (local)	VSMC	???	[131]
miR-221/−222	Detrimental	Rat carotid artery balloon injury model – miR antagomir (local)	VSMC/EC	CDKN1B/CDKN1C	[135]
miR-329	No effect	Mouse femoral artery cuff model – miR antagomir (local)	???	???	[138]
miR-424	Beneficial	Rat carotid artery balloon injury model – miR adenoviral over-expression (local)	VSMC	CCND1 & CALU	[137]
miR-494	No effect	Mouse femoral artery cuff model – miR antagomir (local)	???	???	[138]
miR-495	Detrimental	Mouse femoral artery cuff model – miR antagomir (local)	VSMC/Mac	???	[138]
miR-663	Beneficial	Mouse carotid artery ligation model – miR adenoviral over-expression (local)	VSMC	JUNB	[139]

#### 4.2.1 *miR-21*

In line with the limited human findings, miR-21 levels demonstrated the greatest increase in expression after vascular injury [112], suggesting a prominent role for this microRNA in the induction of neointimal formation. In accordance, several studies have demonstrated that depletion of miR-21 suppresses neointimal formation after angioplasty [112], stent deployment [109,114], or vein grafting [108]. VSMCs have been ascribed as the primary source and modulated cell type by miR-21, suppressing VSMC proliferation and migration through modulation of PTEN [108,112,114], BMPR2 [108] and BCL2 [112]. Although within the in-stent restenosis models, miR-21 was also proposed to affect macrophage invasion and polarisation, possibly through targeting of PPARγ [109].

#### 4.2.2 *miR-15/16*

Two members of the miR-16 family, miR-15b/-16, are highly conserved amongst mammalian species and have been shown to be down-regulated during VSMC phenotypic switching from contractile to a synthetic phenotype in vitro and in vivo [115]. The miR-15b/-16 modulation of VSMC differentiation was shown to be through direct regulation of YAP [115], a key regulator of VSMC phenotypic modulation [116]. Accordingly, local adenoviral-mediated over-expression of miR-15b/-16 suppressed VSMC phenotypic switching, proliferation and migration, resulting in reduced neointimal formation, all associated with decreased YAP expression [115].

#### 4.2.3 *miR-23*

Similarly, miR-23b has been identified as a regulator of VSMC phenotypic switching through direct regulation of FOXO4, and local adenoviral-mediated over-expression of miR-23b reduced neointimal formation within balloon-injured rat carotid arteries [117].

#### 4.2.4 *miR-24*

A beneficial role for miR-24 has also been demonstrated as adenoviral over-expression limited neointimal formation after carotid artery balloon injury in diabetic rats, associated with an anti-proliferative effect of miR-24 linked to modulation of a WNT4-dependent signalling pathway [118].

#### 4.2.5 *miR-26*

Over-expression of miR-26 also reduces VSMC proliferation and migration through targeting MAPK6, and inhibited neointimal formation in a rat model of autologous jugular vein grafting [119].

#### 4.2.6 *miR-29*

Likewise, over-expression of miR-29 using local oligonucleotide delivery to the carotid artery suppressed balloon injury-induced neointimal formation [120]. In vitro analysis revealed that IL-3 stimulated VSMC proliferation and migration were associated with decreased miR-29b expression, and subsequent restoration of miR-29b levels blunted VSMC growth through regulation of Mcl1 and MMP-2 expression [120].

#### 4.2.7 *miR-30*

Intraluminal lentiviral delivery of miR-30 inhibited neointimal formation in rats, attributed to suppression of CaMKIIδ protein expression [121]. Indeed, niR-30 was shown to directly target CaMKIIδ and suppress rat VSMC proliferation and migration [121], in agreement with a deleterious role for CaMKIIδ in VSMC growth and neointimal formation [122].

#### 4.2.8 *miR-34*

Two members of the miR-34 family, miR-34a and miR-34c, have both been demonstrated to play protective roles in injury-induced neointimal formation [123,124]. VSMC proliferation and migration were perturbed by over-expression of miR-34a and miR-34c, through direct regulation of Notch1 [123] and SCF [124] respectively. Of note, despite multiple lines of evidence showing a role for miR-34a in the regulation of endothelial cell behaviour, re-endothelialisation was not assessed in the aforementioned studies, which is of concern given that a wire-injury endothelial denudation model was used [123], and aberrant neointimal formation is attributed in part to delayed re-endothelialisation [125].

#### 4.2.9 *miR-92*

Relatedly, antagomir-directed inhibition of miR-92 enhanced re-endothelialisation of rat carotid arteries after balloon injury or stenting, and consequently reduced neointimal formation [126]. The effects were purported to be endothelial cell specific as proliferation and migration of VSMCs were not affected by miR-92 modulation, and while miR-92 regulated KLF4 and MKK4 expression in endothelial cells, no change was detected in VSMCs [126].

#### 4.2.10 *miR-126*

Recent evidence has demonstrated that endothelial cells undergoing apoptosis release microparticles which diminish VSMC proliferation and neointimal formation in mice [127]. Moreover, endothelial cell-derived microparticles contain abundant miR-126 which is readily transferred to VSMCs in vitro and modulate their behaviour [127]. Furthermore, miR-126 over-expressing microparticles (derived from human coronary artery endothelial cells treated with a miR-126 mimic) reduced neointimal formation in a mouse wire-mediated carotid artery injury model, attributed to suppressed expression of the miR-126 target LRP6 and consequent dampening of the β-catenin signalling pathway [127]. Finally, high miR-126 expression in circulating microparticles (of undefined origin) was associated with reduced rate of coronary revascularization in patients with angiographically-defined coronary artery disease, although the study did not delineate between beneficial effects on atherosclerosis or restenosis [127]. Indeed, in support of an atherosclerosis-dependent effect, a similar study demonstrated circulating microparticles from patients with coronary heart disease have lower miR-126 levels than those obtained from healthy subjects [128].

#### 4.2.11 *miR-132*

Despite up-regulation of miR-132 in rat carotid arteries subjected to balloon injury, delivery of a miR-132 mimic was shown to repress neointimal formation, through attenuation of VSMC proliferation and associated down-regulation of the validated target LRRFIP1 [129].

#### 4.2.12 *miR-133*

A key mechanistic role for miR-133 in the regulation of VSMC phenotypic switching and growth has been shown, through the suppression of the transcription factor Sp-1 [130]. Accordingly, adenoviral-mediated over-expression of miR-133 reduces but miR-133 inhibition aggravates VSMC proliferation and neointimal formation [130].

#### 4.2.13 *miR-143/-145*

Similarly, the miR-143/-145 gene cluster has also been shown to regulate VSMC phenotypic switching and proposed to retard the VSMC differentiation associated with cardiovascular diseases [131,132]. Indeed, over-expression of miR-143 or miR-145 blunted balloon injury-induced neointimal formation in rats, related to suppression of KLF5 expression and preservation of the contractile VSMC markers Acta2 and Myh11 [131,132].

#### 4.2.14 *miR-195*

Over-expression of miR-195 also reduced neointimal formation in the rat balloon-injury model [133]. The proposed beneficial effects of miR-195 were attributed to repression of VSMC proliferation, migration, and expression of growth-related miR-195 target genes Cdc42 and Ccnd1, alongside up-regulation of pro-inflammatory molecules such as IL-1β, IL-6 and IL-8, all determined in vitro upon oxLDL treatment of VSMCs [133].

#### 4.2.15 *miR-206*

Another microRNA identified to regulate VSMC phenotypic switching is miR-206, which is associated with and promotes differentiation to a synthetic VSMC phenotype [134]. As such, lenti-viral over-expression of miR-206 promoted neointimal formation in balloon injured rat carotids, while miR-206 inhibition suppressed it [134].

#### 4.2.16 *miR-221/-222*

During neointimal formation in rats after vascular injury, expression of miR-221 and miR-222 are upregulated, and in vitro studies demonstrated that VSMC proliferation is reduced through knockdown of miR-221 and -222 and associated up-regulation of their direct targets p27 and p57 [135]. Accordingly, downregulation of miR-221 and miR-222 through local adventitial delivery of a combined miR-221/-222 inhibitor, decreased vessel wall VSMC proliferation and neointimal formation in rat carotid arteries subjected to balloon injury [135]. Intriguingly, miR-221 and miR-222 exert divergent functional effects on differing vascular cells. While miR-221 and miR-222 exert pro-proliferative, pro-migratory, and pro-survival effects on VSMCs, they induce opposite roles on endothelial cells [136]. Accordingly, inhibiting miR-221 and miR-222 would yield a dual beneficial role after vascular injury through the retardation of VSMC growth alongside promoting reendothelialization.

#### 4.2.17 *miR-424*

During human VSMC proliferation and neointimal formation in rats, expression of miR-424 (or its rat ortholog miR-322) is decreased [137]. Accordingly, forced over-expression of miR-424/-322 attenuated VSMC proliferation, migration and differentiation in vitro, and limited neointimal formation after balloon angioplasty in rats [137]. The cell cycle regulator cyclin D1 and the calcium-binding protein calumenin which is involved in protein folding and sorting, were both confirmed as direct targets of miR-424/-322 [137].

#### 4.2.18 *miR-495*

Studies using a non-constrictive cuff model in mice, demonstrated that miR-495 knockdown with a gene silencing oligonucleotide suppressed neointimal formation within the femoral arteries [138]. Assessment of the vessel wall revealed that miR-495 silencing reduced the number of medial VSMCs undergoing proliferation, and neointimal macrophage accumulation, although no direct targets of miR-495 were identified to elucidate the underlying mechanisms for these favourable effects [138].

#### 4.2.19 *miR-663*

Finally, miR-663 has been shown to regulate VSMC phenotypic switching as over-expression of miR-663 heightens expression of contractile VSMC genes and subsequently suppresses their proliferative and migratory capacity [139]. Moreover, adenoviral over-expression of miR-663 in a mouse carotid ligation model limits neointimal formation, attributable to direct down-regulation of the key growth-related transcription factor JunB [139]. While the aforementioned studies largely demonstrate biological effects of miR modulation in vivo, there are several studies which have shown redundant roles for a number of microRNAs in neointimal formation, including; miR-1 [130], miR-208 [131], miR-329 [138], and miR-494 [138].

Taken together, the large number of published studies over a short time period demonstrate the appeal of modulating microRNA expression as a therapeutic strategy to limit restenosis after stent deployment or coronary artery bypass grafting (summarised in Fig. 1). The mechanistic in vitro studies and complimentary in vivo animal studies have revealed numerous microRNAs which can be modulated to exert direct effects on VSMC proliferation, migration and phenotypic modulation after vascular injury. Moreover, ancillary findings have shown that some microRNA which limit VSMC growth can exert favourable effects on endothelial regeneration, placing such microRNA at the front of potential therapeutic targets. Unfortunately, due to the limited retrieval of human material after failed stenting or bypass surgery consequent to restenosis, there is a lack of validative data to support the proof-of-concept findings obtained from the numerous animal studies. Nonetheless, the suggestion that plasma microRNA levels or their quantification within circulating microparticles can reflect the biological processes occurring within the vessel wall, could be exploited in humans to predict restenosis rates and the need for further revascularisation. However, many such patients will have extensive atherosclerosis and careful interrogation of causality of specific microRNA apportioned to restenosis or atherosclerotic plaque progression would be necessary.

## 5 Pulmonary arterial hypertension

### 5.1 Human studies

Pulmonary arterial hypertension (PAH) is a particularly severe form of pulmonary hypertension which includes several closely related pathologies characterised by pulmonary arterial endothelial cell dysfunction, progressive growth of the underlying VSMCs, and subsequent medial and neointimal thickening [11]. As such, there are striking similarities in the pathogenesis of PAH with restenosis after vascular injury, including excessive VSMC proliferation and neointimal formation. As with other cardiovascular diseases and cancers, there has been growing interest in the contribution of microRNA in the pathogenesis, diagnosis, and treatment of PAH. Numerous studies have examined resident lung cells or tissues biopsies from PAH patients for dysregulation of microRNAs through unfocussed microarrays or more specifically by evaluating expression levels of individual microRNA. Such studies have identified a number of microRNA which are up-regulated in lung tissues of patients with PAH (predominantly idiopathic PAH) compared to non-diseased individuals, including; miR-let7a [140], miR-21 [141,142], miR-26 [140], miR-27 [140], miR-130 [143], miR-145 [144], miR-199 [140], miR-221 [145], miR-424 [146], and miR-656 [140]. Conversely, decreased expression of miR-21 [147], miR-98 [148], miR-124 [149], miR-140 [150], miR-204 [151], and miR-223 [152] have been reported. In addition, assessment of circulating microRNA levels in PAH patients have revealed changes associated with disease outcome, and therefore purported as potential serum biomarkers of PAH pathogenesis. Elevation of plasma miR-574 levels alongside down-regulation of miR-150 were reported in treatment-naïve PAH patients, and in addition, decreased circulating levels of miR-150 precited poorer survival over time [153]. In line with heightened tissue expression, increased plasma levels of miR-21 [147], miR-23 [154], miR-130 [143,155], miR-133 [155], miR-191 [155], miR-204 [155], miR-208 [155], and miR-301 [143] are also observed in PAH patients. While decreased circulating levels of miR-1 [155], miR-26 [155,156], miR-29 [155], miR-34 [155], miR-451 [155], and miR-1246 [155] have been reported in PAH patients. Finally, a large number of studies have also assessed the effects of PAH-related stimuli on regulation of microRNA expression in pulmonary artery endothelial cells and VSMCs, identifying a number of pertinent microRNAs, their potential targets, and their subsequent effects on cell behaviour (as reviewed by [157]).

### 5.2 Animal studies

Despite the associated caveats with animal models of disease, several rodent models have been designed to mimic the pathogenesis of PAH in humans [158], and subsequently used to examine the expression and contributory roles of select microRNAs to critical features of PAH (summarised in Table 4). Indeed, microarray analysis of lungs from a monocrotaline-induced PAH rat model revealed numerous down-regulated microRNA when compared to control animals; miR-29, -125, -148, -192, -193, -210, -224, -326, -328, -330, -339, -342, -532, -652, -667 and -3557 [159]. Moreover, signalling pathway analysis revealed the TGFβ transduction pathway as a prominent target of the identified down-regulated microRNAs [159], in agreement with a role for dysregulated TGFβ signalling in PAH [160].

**Table 4:** Results of in vivo animal studies evaluating the effect of modulating select microRNA on pulmonary arterial hypertension (PAH).

miRNA(s)	Role	Experimental model – method of microRNA modulation	Cellular origin	Target mRNA	References
miR-17	Detrimental	Chronic hypoxia-induced mouse model & monocrotaline-induced – miR antagomir	VSMC	CDKN1A	[161]
		Decreased pulmonary vascular remodelling & reduced right ventricular systolic pressure (RVSP)			
miR-20	Detrimental	Chronic hypoxia-induced mouse model – miR antagomir	VSMC	BMPR2	[162]
miR-21	Detrimental	Chronic hypoxia-induced mouse model – miR antagomir – decreased vasc pulm remodelling	VSMC	BMPR2, DDAH1, RHOB, PDCD4	[163]
	Detrimental	Chronic hypoxia-induced mouse model – miR antagomir – decreased vasc pulm remodelling & RSVP	VSMC		[142]
	Beneficial	Chronic hypoxia-induced mouse model – miR knockout	EC		[141]
	Beneficial	Chronic hypoxia-induced mouse model – miR knockout & miR over-expression	EC		[164]
	No effect	Chronic hypoxia-induced mouse model – miR antagomir	VSMC		[161]
miR-27	Detrimental	Monocrotaline-induced mouse model – miR antagomir	EC	PPARG	[165]
miR-34	Beneficial	Chronic hypoxia-induced rat model – miR mimic	VSMC	PDGFRA	[166]
miR-96	Beneficial	Chronic hypoxia-induced mouse model – miR mimic	VSMC	HTR1B	[167]
miR-130/301	Detrimental	Chronic hypoxia-induced mouse model – miR antagomir	VSMC/EC	PPARG	[143]
miR-140	Beneficial	Monocrotaline-induced rat model – miR mimic	VSMC	SMURF1	[150]
miR-143	Detrimental	Chronic hypoxia-induced mouse model – miR knockout & miR antagomir	VSMC	???	[171]
	No effect	Chronic hypoxia-induced mouse model – miR antagomir	VSMC		[144]
miR-145	Detrimental	Chronic hypoxia-induced mouse model – miR knockout & miR antagomir	VSMC	???	[144]
miR-204	Beneficial	Monocrotaline-induced rat model – miR mimic	VSMC	SHP2	[151]
miR-210	Detrimental	Chronic hypoxia-induced mouse model – miR knockout & miR antagomir	EC	???	[172]
miR-221	Detrimental	Chronic hypoxia-induced rat model – miR antagomir	VSMC	AXIN2	[145]
miR-223	Beneficial	Monocrotaline-induced rat model – miR mimic	VSMC	PARP1	[152]
miR-424	Beneficial	Monocrotaline-induced rat model, and chronic hypoxia-induced rat model – miR lentiviral over-expression	EC	FGF2, FGFR1	[170]
miR-451	Detrimental	Chronic hypoxia-induced mouse model – miR antagomir	VSMC	???	[173]
	No effect	Chronic hypoxia-induced mouse model – miR knockout	VSMC	???	
miR-503	Beneficial	Monocrotaline-induced rat model, and chronic hypoxia-induced rat model – miR lentiviral over-expression	EC	FGF2, FGFR1	[170]

#### 5.2.1 *miR-17*

A study using both the chronic hypoxia-induced and monocrotaline-induced mouse models demonstrated that miR-17 inhibition, achieved through use of a selective antagomir, reduced pulmonary vascular remodelling and decreased right ventricular systolic pressure (RSVP), indicating a deleterious role for miR-17 in PAH, proposed to be through down-regulation of p21 expression and concomitant increased proliferation of pulmonary VSMCs [161].

#### 5.2.2 *miR-20*

Similar findings have also been reported for miR-20, where antagomir-directed inhibition prevented pulmonary vascular remodelling through restoration of VSMC BMPR2 levels, signalling and associated repression of their proliferation [162].

#### 5.2.3 *miR-21*

Disparate roles for miR-21 in PAH have been proposed, in part reliant on conflicting effects on VSMCs and endothelial cells. Two studies have shown that inhibition of miR-21 protects mice from developing PAH, associated with preservation of TGFβ signalling in VSMCs through restored expression of key proteins such as BMPR2 [142,163]. Conversely, two reports using miR-21 knockout mice [141,164] or over-expression [164] supported a protective role for miR-21 in PAH by promoting endothelial cell survival and vasodilation through repressing PDCD4 [164] and RhoB expression [141], respectively. Supportingly, both human lung tissue and circulating levels of miR-21 have been shown to be reduced in patients with idiopathic PAH [147]. Finally, miR-21 inhibition using an antagomir had no effect in a chronic hypoxia-induced model [161].

#### 5.2.4 *miR-27*

Pulmonary endothelial cell function and vasodilatory capacity has been shown to be regulated by miR-27 due to its direct targeting of PPARγ and associated perturbed NO signalling [165]. Accordingly, miR-27 inhibition ameliorates monocrotaline-induced endothelial dysfunction and consequent PAH in a rat model [165].

#### 5.2.5 *miR-34*

Lung expression of miR-34 is diminished during hypoxia induced PAH in rats, and in isolated pulmonary VSMCs from diseases rats and humans [166]. Restoration of miR-34 levels through intratracheal nebulisation with synthetic miR-34 RNA mimic molecules reversed hypoxia-induced PAH in rats, attributed to down-regulation of VSMC PDGFRA expression and associated perturbation of their proliferation [166].

#### 5.2.6 *miR-96*

Levels of miR-96 are also reduced within the lungs of mice and humans with PAH, particularly females [167]. Relatedly, the incidence of PAH is 3-to-4-fold higher in females than males, suggesting female sex hormones such as oestrogen may exert causative roles in PAH development [168]. Indeed, the addition of oestrogen in the form of 17β-estradiol reduced human pulmonary artery VSMC miR-96 expression while lung miR-96 levels are increased in oestrogen-depleted female mice [167]. Accordingly, tail vein delivery of a miR-96 mimic prevented PAH development in a mouse hypoxia model, associated with down-regulation of the miR-96 target HTR1B, which has previously been shown to promote VSMC proliferation and PAH formation [169].

#### 5.2.7 *miR-130/-301*

A systems biology approach to evaluate key microRNA networks associated with PAH progression identified the miR-130/-301 family as a potential master regulator co-ordinated through regulation of cellular proliferation [143]. Confirmatory studies in mouse, rat and sheep PAH models demonstrated miR-130/-301 family expression is induced during disease development, while inhibition of miR-130/-301 using intrapharyngeal delivery of a specific antagomir halted PAH development in a mouse hypoxia model [143]. Further evaluation revealed the miR-130/-301 family down-regulates PPARγ expression and subsequently suppresses miR-204 and miR-424/503, which exert anti-proliferative effects on pulmonary artery SMCs and ECs respectively [143]. Supportingly, miR-204 expression is decreased in human and mouse experimental PAH and its restoration through delivery of synthetic miR-204 reduced disease severity in a rat monocrotaline model, apportioned to reduced SMC proliferation and their susceptibility to apoptosis [151]. Similarly, levels of miR-424 and miR-503 are reduced in PAH and reconstitution in two experimental rat models prevented and rescued PAH development [170]. Mechanistic studies revealed that apelin regulates the expression of miR-424 and miR-503, and during PAH formation all three are down-regulated in pulmonary artery ECs and is associated with increased expression of FGF2 and FGFR1, promoting proliferation of pulmonary artery ECs and SMCs [170].

#### 5.2.8 *miR-140*

Evaluation of circulating microRNA expression in patients with PAH or other forms of pulmonary hypertension consistently revealed miR-140 expression is decreased, and validative findings in two rodent experimental models also showed reduced miR-140 levels within diseased lung tissues [150]. Treatment with nebulised miR-140 mimic prevented and rescued experimental PAH in both the rat monocrotaline-induced and hypoxia-induced models [150]. A regulator of BMP signalling in pulmonary artery VSMCs, SMURF1, was identified as a miR-140 target, and in accordance with the miR-140 rescue experiments, SMURF1 deletion prevented PAH development in the hypoxic rat model [150].

#### 5.2.9 *miR-143/-145*

The expression of miR-143 is upregulated during PAH in humans and animal models, possibly in a TGF-β1-dependent manner, and has been proposed to promote pulmonary artery VSMC migration [171]. Furthermore, pulmonary artery VSMC-derived exosomes have been shown to exert paracrine pro-migratory and pro-angiogenic effects on pulmonary artery ECs in vitro, although the mechanism and direct target(s) of miR-143 within the ECs is yet to be elucidated [171]. Accordingly, pharmacological inhibition or genetic ablation of miR-143 in mice prevented the development of hypoxia-induced PAH [171]. In contradiction, a similar study by the same group reported that substantial down-regulation of miR-143 expression using a specific miR-143 antagomir had no effect on PAH development, using the same hypoxia-induced mouse model [144], although the authors propose this disparity is due to a lack of statistical power because of small group sizes in the earlier study [171]. However, using similar group sizes, Caruso and colleagues were able to show that anti-miR-145 delivery or miR-145 deficiency protected mice from hypoxia-induced PAH, and demonstrated that BMPR2/TGF-β signalling may regulate the aberrant miR-145 expression observed during PAH [144].

#### 5.2.10 *miR-210*

The expression of miR-210 is elevated in pulmonary artery endothelial cells and the plasma of humans with PAH and experimental mouse models [172]. As expected, genetic ablation of miR-210 or antisense inhibition of miR-210 specifically in vascular endothelium protects mice from hypoxia-induced PAH development [172].

#### 5.2.11 *miR-221*

Recent evidence has shown that miR-221 is elevated within lung samples and isolated pulmonary artery VSMCs from PAH patients and animal models of PAH, and miR-221 directly targets a negative regulator of the β-catenin signalling pathway AXIN2, to promote VSMC proliferation [145]. Consequently, inhibition of miR-221 attenuates the progression of hypoxia-induced PAH in rats [145].

#### 5.2.12 *miR-223*

A protective role for miR-223 in PAH has been advocated as miR-223 expression is reduced within the lungs of patients with PAH and isolated pulmonary artery VSMCs [152]. Indeed, restoration of miR-223 levels through delivery of a miR-223 mimic into the lungs of rats with monocrotaline-induced PAH reversed the disease [152], and this beneficial effect was associated with down-regulation of the miR-223 target PARP1 and therefore attenuating VSMC proliferation and survival [152].

#### 5.2.13 *miR-451*

Finally, in vivo findings have shown that acute suppression of miR-451 with a specific antagomir retarded disease severity in a hypoxia-induced PAH rat model, attributed to reduced pulmonary artery VSMC migration [173]. However, the same study also reported that chronic genetic ablation of miR-451 in mice has no beneficial effect, which the authors suggest may be due to pathway redundancy compensating for long-term miR-451 loss [173].

As can be observed from above, there has been a notable number of studies evaluating the expression patterns and functional roles of microRNAs in PAH. Using primarily rodent models of PAH induced by hypoxia or monocrotaline administration, specific microRNAs have been identified which can exert protective effects and those that display deleterious properties (summarised in Fig. 1). Moreover, knowledge has been gained into which cells produce certain microRNAs and the signalling pathways which regulate their expression. Indeed, through such approaches, the bone morphogenic gene and other TGF-β superfamily members alongside the PPARγ and apelin pathways appear to be notable key networks in the pathogenesis of PAH, through either their regulation of select microRNAs or reciprocal modulation by explicit microRNAs. Furthermore, identification and validation of key targets of microRNAs which are common to deleterious biological processes in PAH, such as uncontrolled VSMC proliferation, may aid stratification of therapeutic desirable microRNAs. However, due to the wide range of predicted targets for each microRNA, this limits the therapeutic approach of using systemic delivery to combat PAH progression. It would prove more efficacious to use a more localised delivery approach such as intranasal application, particularly to target or over-express endothelial microRNA [170]. Therefore, the identification of a key microRNA in combination with the ideal delivery method may prove a positive approach for the development of microRNA-based therapies to prevent PAH progression.

## 6 Conclusions

**Fig. 2:**
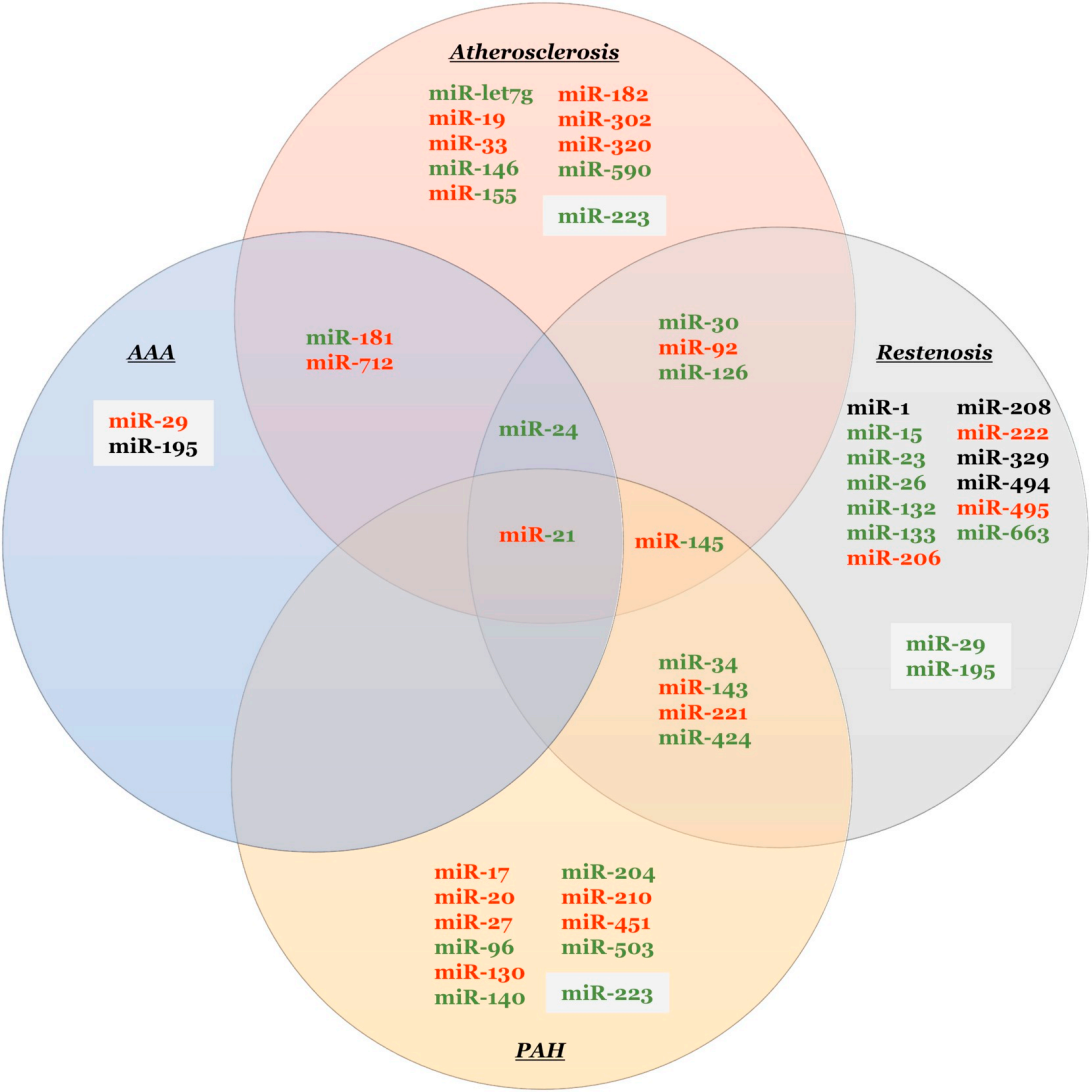
MicroRNA identified in animal studies to exert beneficial or detrimental effects on cardiovascular diseases. This diagram illustrates the microRNA determined detrimental (red), neutral (black), or beneficial (green) in animal models of atherosclerosis, restenosis, pulmonary arterial hypertension (PAH), or abdominal aortic aneurysm (AAA). MicroRNA depicted by two colours have shown differing effects, while microRNA in grey boxes are common between AAA and restenosis or atherosclerosis and PAH.

Over the last decade there has been a large body of published work describing the regulatory role of both specific and clusters of related microRNA exert on the function of vascular and inflammatory cells, alongside effects on cardiovascular pathologies using animal models. In association with human pathological and clinical findings, it is now clear that microRNAs play key roles in a number of cardiovascular diseases and that their modulation may be exploited for therapeutic purposes. Although possibly through a biased approach, several key processes are identified to be under direct microRNA regulation during disease progression, notably, lipoprotein homeostasis, regulation of endothelial cell inflammation, modulation of inflammatory cell recruitment and activation, maintenance of VSMC function and phenotype, and dysregulated proteolysis. These functions play prominent roles in the pathogenesis of atherosclerosis, aneurysms, neointimal formation and PAH, and unsurprisingly, select microRNA have been identified to affect more than one cardiovascular disease (see Fig. 2). For example, miR-21 is increased in human vessels harbouring atherosclerosis, AAA, restenosis or PAH-related plexi-form lesions, suggesting an unfavourable role for this microRNA in the progression of these cardiovascular diseases. However, modulating miR-21 expression in relevant animal models has revealed a protective role for miR-21 in atherosclerosis and AAA, while a detrimental role has been proposed in restenosis, whereas PAH strategies to control miR-21 levels have provided protective, neutral, and detrimental effects. Such findings demonstrate the requirement to consider both off-target effects of a specific microRNA but also effects on other pathologies a patient may be possessing and may consequently exert deleterious effects. For instance, limiting VSMC growth may provide an effective strategy to retard intimal growth after stenting or vein graft implantation, but within an atherosclerotic plaque can impede stability by hindering maintenance of the protective fibrous cap. Similar discrepancies have been noted for miR-145 with regards to atherosclerosis and restenosis, probably through mis-interpretation of findings from systemic interventions and/or analysis of multi-cellular tissues. Indeed, underpinning research has shown that miR-145 plays a prominent role in regulating VSMC phenotypic modulation and consequent function, which may exert divergent effects on atherosclerosis and restenosis, where VSMC growth is beneficial and detrimental respectively. In fact, it is important to consider and elucidate the cell-type(s) responsible for alterations in microRNA expression when conducting whole tissue analysis, to ensure the correct extrapolation and interpretation of the findings, especially biological connections between specific microRNA and potential target genes [72].

Nonetheless, as microRNAs can target multiple genes within common regulatory networks, controlling microRNAs may be exploited to effect biological functions and pathways within diseased arteries and other related organs such as the liver, myocardium and spleen. Furthermore, utilising a custom selection of microRNA inhibitors and/or mimics may provide an appealing therapeutic approach to manage single and co-existing cardiovascular diseases and their clinical complications. Further improvements in delivery strategies will also prove favourable, including cell-targeted delivery, as recently demonstrated using microRNA-containing microparticles enriched with miR-146a and miR-181b [174]. Such cell type-specific strategies will be necessary to exploit the therapeutic potential of several promising anti-atherosclerotic approaches to modulate select microRNA, such as targeting miR-33 solely in macrophages to ensure potentially damaging off-target effects within the liver are negated. Although no microRNA-based therapeutics have advanced into clinical testing for cardiovascular pathologies, several have reached clinical development for other diseases [175]. Notably, modified antisense inhibitors of miR-122 for hepatitis C, and chemically-enhanced mimics to over-express miR-16, miR-29, or miR-155 to target differing forms of cancer [175]. These developments hopefully pave the way for clinical studies utilising microRNA therapeutics in cardiovascular diseases but will require identification of the most promising key candidate microRNA or microRNA targets for each individual disease type. Furthermore, refinement and further development of novel delivery and cell-targeting platforms will avoid potential off-target effects and toxicities and facilitate the use of microRNA therapeutics in the cardiovascular clinical arena.

## Sources of funding

Dr. Johnson is the recipient and funded through a British Heart Foundation Senior Basic Science Research Fellowship (FS/18/1/33234).
